# Screening and Replication using the Same Data Set: Testing Strategies for Family-Based Studies in which All Probands Are Affected

**DOI:** 10.1371/journal.pgen.1000197

**Published:** 2008-09-19

**Authors:** Amy Murphy, Scott T. Weiss, Christoph Lange

**Affiliations:** 1 Channing Laboratory, Brigham and Women's Hospital, Boston, Massachusetts, United States of America; 2 Harvard Medical School, Boston, Massachusetts, United States of America; 3 Center for Genomic Medicine, Brigham and Women's Hospital, Boston, Massachusetts, United States of America; 4 Department of Biostatistics, Harvard School of Public Health, Boston, Massachusetts, United States of America; University of Alabama at Birmingham, United States of America

## Abstract

For genome-wide association studies in family-based designs, we propose a powerful two-stage testing strategy that can be applied in situations in which parent-offspring trio data are available and all offspring are affected with the trait or disease under study. In the first step of the testing strategy, we construct estimators of genetic effect size in the completely ascertained sample of affected offspring and their parents that are statistically independent of the family-based association/transmission disequilibrium tests (FBATs/TDTs) that are calculated in the second step of the testing strategy. For each marker, the genetic effect is estimated (without requiring an estimate of the SNP allele frequency) and the conditional power of the corresponding FBAT/TDT is computed. Based on the power estimates, a weighted Bonferroni procedure assigns an individually adjusted significance level to each SNP. In the second stage, the SNPs are tested with the FBAT/TDT statistic at the individually adjusted significance levels. Using simulation studies for scenarios with up to 1,000,000 SNPs, varying allele frequencies and genetic effect sizes, the power of the strategy is compared with standard methodology (e.g., FBATs/TDTs with Bonferroni correction). In all considered situations, the proposed testing strategy demonstrates substantial power increases over the standard approach, even when the true genetic model is unknown and must be selected based on the conditional power estimates. The practical relevance of our methodology is illustrated by an application to a genome-wide association study for childhood asthma, in which we detect two markers meeting genome-wide significance that would not have been detected using standard methodology.

## Author Summary

The current state of genotyping technology has enabled researchers to conduct genome-wide association studies of up to 1,000,000 SNPs, allowing for systematic scanning of the genome for variants that might influence the development and progression of complex diseases. One of the largest obstacles to the successful detection of such variants is the multiple comparisons/testing problem in the genetic association analysis. For family-based designs in which all offspring are affected with the disease/trait under study, we developed a methodology that addresses this problem by partitioning the family-based data into two statistically independent components. The first component is used to screen the data and determine the most promising SNPs. The second component is used to test the SNPs for association, where information from the screening is used to weight the SNPs during testing. This methodology is more powerful than standard procedures for multiple comparisons adjustment (i.e., Bonferroni correction). Additionally, as only one data set is required for screening and testing, our testing strategy is less susceptible to study heterogeneity. Finally, as many family-based studies collect data only from affected offspring, this method addresses a major limitation of previous methodologies for multiple comparisons in family-based designs, which require variation in the disease/trait among offspring.

## Introduction

Recent advances in mapping array technology and the increasing content from SNP databases [1],[2] have expanded the capacity for large-scale genotyping. With mapping arrays for more than one million SNPs now available [3],[4],[5],[6], genome-wide association studies carry the promise of identifying replicable associations between important genetic risk factors and complex diseases. One of the major hurdles that needs to be addressed in order to make genome-wide association studies successful is the multiple comparison problem. Hundreds of thousands of SNPs are genotyped and examined for potential associations with multiple phenotypes, resulting in possibly millions of statistical tests. The small number of SNPs that contain “true” signals must be identified among the thousands of false-positive results. The success of genome-wide association studies will depend upon whether it will be possible to overcome this obstacle and translate the increase in genotype information into the identification of novel disease loci, or whether the increased genetic information will be diluted by the multiple testing problem.

A brute-force way to address the multiple comparison problem is to design studies with sample sizes large enough to test all genotyped SNPs with standard association tests and adjust for multiple comparison using the Bonferroni correction [7]. However, while sample sizes of several thousand subjects will certainly be feasible for common phenotypes (e.g., BMI, height), such a strategy carries the risk that the increase in sample size is accompanied by an increase in study heterogeneity, mitigating the positive effects of a larger sample size. Further, for many diseases, recruiting the theoretically required sample size may not be feasible, prohibited either by the costs for recruitment or phenotype assessment, or by the prevalence of the disease. An alternative approach is to develop novel statistical methodology to address the multiple comparison problem with realistic sample sizes.

For the analysis of quantitative traits in family-based designs, Van Steen et al. [8] proposed a new class of two-stage testing strategies that uses the same data set twice, first for genomic screening and then for genetic association testing. The approach proved to be a very powerful way to address the multiple testing problem in genetic association studies [8],[9],[10],[11]. Van Steen type testing strategies take advantage of a unique property of family-based data in that it can be partitioned into two statistically independent components. By exploiting the information about the genetic association that is not used in the second stage when the association tests are computed, the first stage prioritizes “promising” SNPs for the second stage.

Van Steen type testing strategies have three key advantages: 1.) The method achieves statistical power levels which can be substantially higher than those of standard approaches [8],[9], and is thereby able to establish genome-wide significance within one study [8],[9],[10],[11]. 2.) The Van Steen algorithm maintains the separation between the multiple testing problem and the replication process. Replication attempts in different studies are reserved for the generalization of the established associations and assessment of heterogeneity between study populations. 3.) Since genome-wide significance is established in the first data set, the number of SNPs that are pushed forward for replication testing in other populations is generally very small and does not require a large budget, which makes simultaneous replication attempts in multiple samples feasible.

Although the approach has recently been significantly improved and now allows family studies to achieve power levels that are comparable to population-based studies with the same number of probands [9], its applicability is limited. While extensions of the testing strategy are available for arbitrary family structures and for case/control designs [10],[11], the approach cannot be applied in situations in which there is no phenotypic variation in the phenotypes of the probands, i.e., all probands are affected with the disease or trait of interest. This prevents the utilization of the approach in trio designs (i.e., affected probands and their parents). Since this original trio/TDT design is frequently used, this limitation of the testing strategy poses a major disadvantage for family-based designs.

In this manuscript, we propose an extension of Van Steen type testing strategies to family-based designs in which all probands are affected. The strategy also uses the same data set for both stages, which we will refer to as the rank-weighting step and the testing step. In the first stage of the testing strategy, the genetic relative risk effect sizes are estimated for each SNP. We show that it is possible to derive four estimating equations that depend only on the observed parental mating types, but not on any unknown parameters. The estimating equations can be solved analytically, allowing for the construction of effect size estimators that do not depend on the marker allele frequency or offspring genotypes. This is in contrast to effect size estimators/association test statistics for study designs with only affected subjects in population-based studies [12],[13],[14], where the allele frequency must be specified.

Based on the genetic effect size estimates obtained from the estimating equations, we compute the conditional power of the FBAT/TDT for all SNPs. The relative rank of the SNPs by conditional power is then used in a weighted Bonferroni approach [9] to assign each SNP an individually adjusted significance level. The weights are constructed so that the overall type-1 error is maintained. In the second step of the testing strategy, the FBAT/TDT statistic is computed for each SNP and genome-wide significance is established based on its individually adjusted significance level.

Using extensive simulation studies, the statistical power of the testing strategy is assessed for over a range of genetic effect sizes, different numbers of trios, when the mode of inheritance is known and unknown, and in the absence and presence of linkage disequilibrium (LD). The practical relevance of the approach is illustrated by an application to a genome-wide association study of childhood asthma.

## Methods

### An Overview of Partitioning Family-Based Data into Independent Components

Van Steen testing strategies for genome-wide association studies partition the data set into two statistically independent, but overlapping parts [8],[9],[10],[11],[15],[16]. In family-based designs, the first component contains information about the SNP-trait association at a population level, which is assessed based on the proband's phenotype, *Y*, and the parental genotypes, *P*_1_, *P*_2_ [15],[17]. In our application, we use the offspring phenotype and parental genotypes to construct effect size estimates of the genetic relative risk. The second component of the data characterizes the SNP-trait association at the family level, i.e., the allele transmissions from the parents to their offspring [18],[19],[20]. Family-based association tests such as the TDT or FBAT are therefore conditional tests that treat the offspring genotype, *X*, as random, conditioning upon the offspring phenotype, *Y*, and the parental genotypes *P*_1_, *P*_2_. The evidence for SNP-trait association is evaluated by comparing the observed offspring genotype with the expected offspring genotype, which are computed by conditioning upon the parental genotypes, assuming Mendelian transmissions. Since the offspring genotype is the only random component of the FBAT/TDT statistic, the implication is that other information in the FBAT/TDT statistic (i.e., the offspring phenotype and parental genotypes) may be used to assess the evidence for association without biasing the significance level of the FBAT/TDT statistic.

Based on the two information sources about association in family-based designs, the density of the joint distribution for *X*, *Y*, and *P*_1_, *P*_2_ can then be partitioned into two statistically independent components [21],
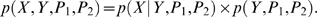
(1)Since the density for the first step of the testing strategy, the rank-weighting step, is given by *p*(*Y*, *P*_1_, *P*_2_), and the density of the second step, the FBAT/TDT testing step, is *p*(*X*|*P*_1_, *P*_2_, *Y*), likelihood decomposition (Equation 1) implies that the two steps of the testing strategy are independent. The “evidence of association” (i.e., the genetic effect size estimate) for each marker from the rank-weighting step can be utilized in the second stage without having to adjust the overall significance level for the estimation of the genetic effect size in the first stage. There are various ways in which the information from the rank-weighting step can inform the application of the FBAT/TDT statistic in the second step. The effect size estimate from the screening step can be used to select a small subset of “very promising” markers for FBAT/TDT testing [8] or to assign each marker with an individual significance level that reflects the rank of the marker's effect size estimate relative to the other markers [9]. Another possibility is to have the information from the screening step define the “tuning parameters” of the FBAT statistic [22],[23].

### The Rank-Weighting Step: Estimating the Power of the FBAT Statistic under *H_A_* When Trio Data Are Given and All Probands Are Affected

We assume that trios are given (i.e., affected probands and parents), and that SNP data are analyzed. If the parental data are missing/unavailable, the parental genotypes can be replaced in all equations below by the sufficient statistic by Rabinowitz & Laird [18],[19]. The sufficient statistic for each nuclear family is defined by all family configurations that lead to consistent inference about the missing parents, given the observed genotypes. When parental data are given, the parental genotypes represent the sufficient statistic. Like the parental genotypes, the sufficient statistic allows for the computation of the offspring genotype distribution within each family, independent of the unknown allele frequency. For a more detailed discussion, we refer to the original paper [18].

For each marker locus of interest, let *x_i_* be the coded genotype of the *i* ^th^ proband, counting the number of minor alleles for the SNP of interest. The variables *p_i_*_1_ and *p_i_*_2_ denote the parental genotypes for both parents at the locus. The phenotype of the *i* ^th^ proband is defined by *y_i_*. For trio samples in which all probands are affected, the phenotype is coded as “y = 1”. The FBAT statistic, 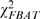, [19],[20] is then given by:
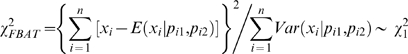
(2)and has a chi-square distribution with one degree of freedom. Assuming an additive coding function for the genotype, this FBAT statistic and the original TDT statistic [20] are equivalent.

In order to develop a Van Steen type testing strategy [15],[16] for the classical TDT design, the conditional power [22],[24] of the FBAT/TDT statistic, 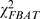, has to be computed in the first step of the testing strategy. This requires the specification of the conditional marker density under the alternative hypothesis:
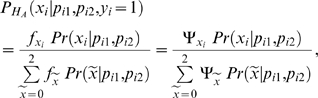
(3)where affected probands are coded as “*y_i_* = 1”. The parameter *f_x_* denotes the penetrance probability (i.e., *f_x_* = *Pr*(*y_i_* = 1|*x*)), and Ψ*_x_*, the genotype relative risk (i.e., Ψ*_x_* = *f_x_*/*f*_0_). The probability *Pr*(*x*|*p_i_*_1_, *p_i_*_2_) is defined by Mendelian transmission and can be computed straightforwardly, conditional on parental genotypes, without any additional knowledge/assumptions. The penetrance probabilities 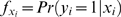 are unknown and have to be estimated based on the information that is available in the rank-weighting step, i.e., the offspring phenotype and the parental genotypes.

In the original Van Steen approach [8], the parental genotypes are used to compute the expected/predicted marker scores of the offspring. By regressing the offspring phenotype on its expected marker score, an estimate for the genetic effect size is obtained that allows us to specify the penetrance probability, *Pr*(*y_i_* = 1|*x_i_*) [15],[16]. However, when there is no phenotypic variation in the data (i.e., all probands are affected), this approach is not applicable and an alternative approach has to be developed. In order to simplify the notation, our derivation will be based on the parameterization of the marker distribution (Equation 3) in terms of the genotype relative risks, Ψ*_x_*.

Due to the lack of variation in the phenotype, the only variation that can be utilized for the estimation of the relative risk probabilities are the parental genotypes. In the trio design, there are six distinct parental mating types: (*p*_1_ = 2, *p*_2_ = 2), (*p*_1_ = 2, *p*_2_ = 1), (*p*_1_ = 2, *p*_2_ = 0), (*p*_1_ = 1, *p*_2_ = 1), (*p*_1_ = 1, *p*_2_ = 0) and (*p*_1_ = 0, *p*_2_ = 0), where 0, 1, and 2 denote the number of copies of the minor allele for the marker of interest. The frequencies of the parental mating types in the ascertained sample (*y_i_* = 1) can be computed using Bayes' rule,
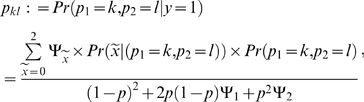
(4)where the parameter, *p*, denotes the minor allele frequency for the marker in the general population, and again, as above, the probabilities 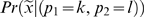 are defined by Mendelian transmissions. The probabilities *Pr*(*p*_1_ = *k*, *p*_1_ = *l*) are the paternal mating type frequencies in the general population, and *k* and *l* are given by one of the six distinct mating types defined above. Under the assumption of random mating and Hardy-Weinberg equilibrium at the marker locus in the general population, the probabilities *Pr*(*p*_1_ = *k*, *p*_1_ = *l*) will be defined by the actual mating type and the minor allele frequency, *p*.

Based on these assumptions, the likelihood of the parental mating types in the ascertained sample is given by 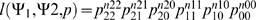, where the probability of a mating type is denoted as 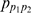 and the observed number of mating types is 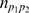. In order to obtain maximum likelihood estimates for the genotype relative risks Ψ_1_ and Ψ_2_, one has to maximize the likelihood function *l*(Ψ_1_, Ψ2, *p*) over all unknown parameters, i.e., the genotype relative risks, Ψ_1_ and Ψ2, and the minor allele frequency of the marker, *p*. However, due to the structure of the likelihood function, the Fisher information matrix is ill conditioned [25] and a numerical solution of the likelihood maximization is non-trivial. This is particularly challenging in the context of genome-wide association studies in which the numerical implementation must be fast and reliable. In addition to the technical issues related to the likelihood maximization, the estimation of the allele frequency at the marker locus is also problematic in the presence of population admixture.

To avoid issues related to the estimation of the allele frequency, we will construct estimators for the genotype relative risks, Ψ_1_ and Ψ_2_, that are independent of the minor allele frequency, *p*, and have a closed analytical form, facilitating a numerically fast and robust implementation in genome-wide association studies. We consider the following four possible ratios of parental mating types:
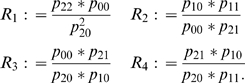
(5)Under the assumption of Hardy-Weinberg equilibrium in the general population, using (Equation 4), the minor allele frequency, *p*, drops out of the mating type ratios, and one can show that the ratios *R*_1_, *R*_2_, *R*_3_, and *R*_4_ are given by:
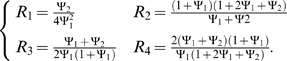
(6)


It is important to note that the four ratios *R*_1_, *R*_2_, *R*_3_, and *R*_4_ do not depend on the unknown minor allele frequency, *p*, and can be estimated based on the parental genotypes, e.g., 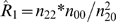. It is also important to note that, if a likelihood approach for the parental mating types had been implemented, the minor allele frequency, *p*, would have to be estimated.

If a genetic model is specified (e.g., under an additive mode of inheritance, Ψ_1_ = (1+Ψ_2_)/2), each equation in (Equation 6) will depend only on one unknown genotype relative risk parameter. Each equation can then be solved for the unknown parameter and four estimates for the genotype relative risk are obtained. Alternatively, an overall effect size estimate can be constructed by averaging over all four estimates for the genetic effect size. The selected estimate for the genotype relative risk can then be used to calculate the marker distribution under the alternative hypothesis (Equation 3), which is the final component needed in calculating the conditional power of the FBAT/TDT statistic. Using simulation studies, we will assess which of the four ratios (or the average) for the proposed testing strategy generally achieves the highest and most stable power estimates.

Since the proposed estimators for the genotype relative risk only depend on the parental genotypes, they fulfill the decomposition condition (Equation 1) and can be used in the rank-weighting step of the testing strategy without biasing the significance level of the FBAT/TDT statistic in the second stage. The independence of the mating type ratios from the allele frequency makes the approach particularly attractive in the presence of population admixture.

While we have outlined the concept of genotype relative risk estimation in the context of ascertained family samples for the trio designs, the genetic effect size estimators can be constructed in the same way for more complex nuclear family structures. Using the algorithm by Rabinowitz & Laird [18], all possible parental mating types can be derived for nuclear families with missing parental information and/or multiple offspring. The mating type probabilities can then be computed based on Bayes' rule, as for the trio design (Equation 4). By examining all possible mating type ratios, the ratios that depend only on the genotype relative risk, but not on the allele frequency, can be identified and used to construct direct estimators of the genetic effect size. While we are not able to provide a general rule of thumb on how to construct mating type ratios that do not depend on the allele frequency other than to evaluate all possible ratios, such ratios appear to exist for most nuclear family-types. Since the identification process of the suitable mating type ratios can be automated by using software packages such as Maple and Mathematica, the proposed concept of genotype relative risk estimation is not specific to the trio design and should be applicable to general nuclear family-types.

It is important to note that the proposed genetic effect size estimators are derived under the assumption of Hardy-Weinberg equilibrium at the marker locus in the general population, but not in the ascertained sample. Since it is common practice to filter out SNPs that are strongly out of Hardy-Weinberg equilibrium when the genotype data are cleaned prior to analysis, only SNPs with mild to moderate violations of the Hardy-Weinberg assumption will reach the association analysis step. The effects of SNPs with Hardy-Weinberg violations on the proposed testing strategy are thereby limited. However, the genetic effect size estimation in the first step will be biased for such SNPs. In the presence of SNPs that are out of Hardy-Weinberg equilibrium and that are not associated with affection status, the proposed testing strategy is likely to have reduced power. If the Hardy-Weinberg assumption does not hold at the disease susceptibility locus (DSL), the power of the proposed testing strategy can be either increased or decreased, depending on whether the signal that is caused by the true genetic effect at the DSL locus is amplified by the Hardy-Weinberg violation or not. Further, it is important to note that, while violations of the Hardy-Weinberg assumption will have an effect on the rank-weighting step, the validity of the FBAT/TDT-testing step and, consequently, the validity of the entire approach will not be affected by departures from Hardy-Weinberg.

### The Testing Step: Testing for Family-Based Association with Weighted Bonferroni Significance Levels

In the first phase of the testing strategy, the genetic effect size estimates for each marker are used to compute the conditional power at each locus, and all markers are ranked by power. A weighted Bonferroni approach [9] is implemented that assigns individual significance levels, denoted as *α_i_*, to each marker locus based on its conditional power ranking. Essentially, *α_i_* is the type 1 error apportioned to the *i* ^th^ test on the basis of its power ranking relative to all of the other tests. The individual significance levels are selected so that the overall significance level is maintained, e.g., 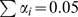. Using the FBAT/TDT statistic, each marker is then tested in the second stage at the individual significance level *α_i_*, and its association with affection status is declared as genome-wide significant if its FBAT/TDT statistic p-value is less than the individual significance level *α_i_*.

In order to determine the individual significance levels *α_i_*, we must select a weighting scheme to apply to the weighted Bonferroni method [9]. Essentially, the weighted Bonferroni method partitions the SNPs into bins and assigns each bin a weight, where the bin and weight sizes vary depending on the relative power ranking of the SNPs in the bin. Each SNP within a bin is assigned an equal weight, which represents a fraction (or individual significance level, *α_i_*) of the overall significance level, *α*. Many different weighting schemes to select bin/weight sizes may be applied, as long as *α* is maintained. We selected an exponential weighting scheme, which uses weights that decrease exponentially and bin sizes that increase exponentially as the power rankings decrease [9]. To define the exponential weighting scheme, let *k_j_* be the size of the *j^th^* partition, and let *k* and *r* be user-defined partitioning parameters with an integer value. Then the sizes of the subsequent partitions can be defined by *k*_1_ = *k* and *k_j_* = k*r ^(*j*−1)^. The exponential weight, *w_j_*, for the *j^th^* bin is given by 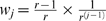, with 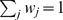. Finally, the individual significance level for the *j^th^* partition/bin is 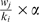. With these parameter specifications, it is straightforward to see that 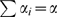, thus the overall alpha level is maintained. Further discussion of the weighted Bonferroni method and weighting schemes is given in Ionita-Laza et al. [9]. The optimal choices for the initial partition size *k* and the partitioning parameter *r* will be determined by simulation studies.

### Simulation Studies

Using simulation studies, we compare the proposed testing strategy to the standard approach, FBAT/TDT testing with Bonferroni corrected p-values. Both approaches are contrasted under various scenarios with differing trio sample sizes and minor allele frequencies. We simulate trio data under the assumption that all offspring are affected and the genotypes of both parents are known. The minor allele frequencies are drawn from *β* distributions that resemble the 550 K Illumina HumanHap array.

The data were simulated under two separate scenarios. In the first scenario, independence among all markers (i.e., no linkage disequilibrium (LD)) is assumed. In the second scenario, we simulated local LD between the SNPs. In order to obtain realistic local LD patterns, we utilized a 550 K scan in the CAMP study (see Data Analysis section) that consists of 400 trios. Based on the observed local LD patterns in CAMP, we simulated the correlated SNPs for the second scenario. Specifically, we applied a ‘moving window’ algorithm, where the observed correlation (*r* ^2^) between the SNP to be simulated and the SNP immediately preceding the SNP that is simulated (in terms of physical location) was used to recapitulate local LD patterns on a genome-wide scale.

In each simulation, one locus/SNP is assumed to be the DSL, while the other SNPs that are not in LD with the DSL are considered null loci. For the null loci, under the independence scenario, the parental genotypes are generated by drawing from a Binomial distribution with the selected marker's minor allele frequency. When SNPs are correlated, the moving window approach described above is used to generate parental genotypes. Based on the parental genotypes, the offspring genotype is obtained by simulated Mendelian transmissions from the parents. At the DSL, the configuration of genotypes in the proband and parents is simulated based on their theoretical distribution under the specified alternative hypothesis, as outlined in Knapp [26] and Lange & Laird [22],[24].

For the considered scenarios, we assessed the performance of the proposed approach when the genetic effect size is estimated either based on one of four mating type ratios (R1–R4, Equation 6) or by the average of the four estimates. In simulation studies comparing the performance of the estimators (data not shown), we observed that the genotype relative risk estimator based on equation R4 consistently generated the highest power estimates (for minor allele frequencies (MAFs) >0.1), and was stable, even with modest effect sizes (e.g., OR = 1.25) and lower allele frequencies (e.g., MAF≤0.2). Thus, all estimated power levels for the proposed method that are shown here are based on the genotype relative risk estimator for mating type ratio R4.

In the first set of simulations, we assume an additive mode of inheritance at the DSL. The genetic effect size is defined in terms of an odds ratio and ranges between 1.25 and 2.5, depending on the number of trios. A disease prevalence (*K*) of 10% is selected throughout the simulations. The trio sample size varies between 500–2000 trios. To accurately depict the degree of LD between markers, 500,000 markers are simulated. Under the independence scenario, the power was assessed as the proportion of replicates where the FBAT test statistic p-value was less than the required weighted Bonferroni alpha level, based on its power ranking from the rank-weighting step. Under the LD scenario, the power was computed in two ways. First, we defined a positive result identically to the procedure used for the independence scenario (i.e., a significant result for the DSL only). Secondly, we more broadly defined a positive result to include a significant finding in the DSL or in any markers in strong LD (*r* ^2^>0.8) and within the same physical region, (i.e., within five SNPs) with the DSL. For the standard Bonferroni correction, power was defined as the proportion of replicates with an FBAT statistic p-value<10^−7^ (i.e., 0.05/500,000).

### Estimated Power Levels for n = 500–2000 Trios, under an Additive Genetic Model

The results of the first set of simulations are displayed in Table 1. The number of trios is presented in column 1 and the odds ratio (OR) for the DSL is specified in column 2. The minor allele frequency (MAF) of the DSL is displayed in Column 3. Columns 4, 6, and 8, denoted as “Weighted,” present the power estimates using the weighted Bonferroni method by Ionita-Laza et al. [9], with an exponential weighting scheme and partitioning parameters of K = 7 and r = 2. The choice of K = 7 and r = 2 tended to have the highest power among a range of partitioning (K = 3–10, r = 2–5) parameters, although decreases in power were minimal within these ranges (data not shown). Columns 5, 7, and 9, denoted as “Standard,” display the results for the standard approach in which all SNPs are equally weighted when applying the Bonferroni correction, and a significance level of 10^−7^, (i.e., 0.05/500,000) is required for genome-wide significance. Columns 4–5 (Independence scenario”) reflect the scenario in which all markers are independent (i.e., adjacent *r* ^2^ = 0). Columns 6–9 (“LD scenario”) display the power estimates when LD is present among markers, where the power represents either detecting the DSL only (Column 6–7), or the DSL/markers in strong LD with the DSL (Columns 8–9). The power estimates are based on at least 1,000 replicates for each (DSL) minor allele frequency and odds ratio.

**Table 1:** Power for 500–2000 trios and 500K markers, using mating type ratio equation R4, under an additive genetic model.

Number	Odds	MAF	Independence scenario		LD scenario (DSL only)		LD scenario (DSL+)	
of Trios	Ratio		Weighted	Standard	Weighted	Standard	Weighted	Standard
**2000**								
	1.25	0.1	0.066	0.003	0.042	0.001	0.127	0.017
		0.2	0.241	0.039	0.168	0.012	0.391	0.147
		0.3	0.295	0.089	0.203	0.031	0.513	0.300
		0.4	0.270	0.129	0.165	0.048	0.504	0.366
	1.375	0.1	0.226	0.078	0.154	0.033	0.371	0.195
		0.2	0.591	0.388	0.454	0.212	0.800	0.665
		0.3	0.744	0.591	0.590	0.397	0.921	0.857
		0.4	0.764	0.666	0.591	0.465	0.930	0.893
	1.5	0.1	0.517	0.357	0.390	0.225	0.722	0.604
		0.2	0.908	0.846	0.827	0.703	0.985	0.964
		0.3	0.976	0.952	0.931	0.874	0.995	0.992
		0.4	0.979	0.969	0.940	0.902	0.997	0.995
**1000**								
	1.5	0.1	0.100	0.032	0.072	0.018	0.170	0.084
		0.2	0.354	0.189	0.271	0.113	0.520	0.352
		0.3	0.470	0.336	0.360	0.220	0.667	0.555
		0.4	0.456	0.371	0.333	0.248	0.660	0.571
	1.75	0.1	0.438	0.324	0.345	0.236	0.581	0.488
		0.2	0.859	0.777	0.770	0.658	0.940	0.901
		0.3	0.932	0.896	0.881	0.819	0.976	0.960
		0.4	0.936	0.904	0.881	0.839	0.976	0.964
	2	0.1	0.825	0.759	0.750	0.669	0.918	0.876
		0.2	0.992	0.985	0.984	0.970	0.999	0.997
		0.3	0.998	0.996	0.994	0.990	1.000	1.000
		0.4	0.997	0.995	0.994	0.989	0.998	0.997
**500**								
	2	0.1	0.184	0.128	0.132	0.085	0.276	0.205
		0.2	0.573	0.480	0.490	0.382	0.693	0.606
		0.3	0.711	0.628	0.635	0.538	0.805	0.740
		0.4	0.665	0.590	0.591	0.505	0.771	0.707
	2.25	0.1	0.447	0.350	0.367	0.278	0.551	0.473
		0.2	0.849	0.787	0.797	0.720	0.916	0.878
		0.3	0.905	0.868	0.869	0.811	0.954	0.928
		0.4	0.894	0.856	0.849	0.805	0.934	0.900
	2.5	0.1	0.694	0.612	0.624	0.542	0.793	0.729
		0.2	0.957	0.934	0.935	0.895	0.981	0.967
		0.3	0.978	0.964	0.966	0.943	0.991	0.982
		0.4	0.965	0.949	0.945	0.919	0.983	0.975

Estimated power levels to detect the DSL using 500–2000 trios, assuming a 10% disease prevalence and additive mode of inheritance. The significance level is set to 5%. For the weighted Bonferroni method (Weighted), the partitioning parameters are *K* = 7 and *r* = 2. MAF denotes minor allele frequency. The power reflects the proportion of times the p-value of the DSL (Independence scenario and LD scenario (DSL only)) or a SNP in LD with the DSL (LD scenario (DSL+)) met the weighted Bonferroni (Weighted) or standard Bonferroni corrected (Standard) significance level. The standard Bonferroni correction adjusts for 500 K comparisons.

For genome screens of 500 K SNPs, regardless of the sample size or degree of correlation among markers, the use of power-driven weights from the rank-weighting step shows a considerable improvement in power over the standard methodology. For the lowest power estimates (<40% power for the standard Bonferroni), the power estimates for the weighted method are typically at least twofold greater than the standard approach. For low to moderate power estimates, (40–70% power for Bonferroni), the weighted method outperforms the standard correction by to 15–40%. For SNPs with greater than 70% power with the standard approach, the improvement ranges between 7 and 11%, unless the power estimates are near one. However, even in these scenarios, the power estimates for the weighted Bonferroni method are always higher, though the differences between the two methods are more modest.

With respect to trio sample size, we note that even with smaller sample sizes (e.g., n = 500), there is still power to detect a DSL (or SNP in LD with the DSL), and the power gains over standard Bonferroni correction are maintained, although a more pronounced effect size is required (OR = 2.25–2.5) to achieve adequate power. Based on the results of our simulation studies, we would not recommend genome-wide association studies of fewer than 300 trios unless extremely large effect sizes (OR>3) were anticipated.

To verify that the proposed testing strategy maintains the overall alpha level, the simulations were repeated under the null hypothesis of no linkage/no association, with a sample size of 500 trios. Based on over 10,000 replicates, the observed overall type 1 error rate was maintained at 4.66%.

Finally, in examining the impact that LD has on power, when considering a positive finding to be the detection of the DSL only, the power of the approach was slightly reduced in comparison to the scenario in which the SNPs were independent. However, the proposed testing strategy still outperforms the standard approach by differences that are of practical relevance. When the definition of a positive finding is extended to those SNPs that are in LD with the DSL, the power estimates are higher than the independence scenario. This is a significant finding, given that some array platforms for genome-wide genotyping do not employ LD-tagging methods, and as chip density increases (i.e., one million SNP arrays), linkage disequilibrium will have a greater impact on the analysis of genome-wide association studies.

### Estimated Power Levels for n = 2000 Trios, When the Genetic Model Is Unknown

Since in practice the underlying mode of inheritance is unknown, we ran a second set of simulations to reflect this reality and assess the impact on the power of the proposed method and the standard approach. In the data analysis step of the following simulation, the true genetic model was considered to be “unknown.” We simulated three scenarios, where the true (but unknown) generating model was either additive, dominant, or recessive, and conducted separate FBAT analyses under all three genetic models. To evaluate the power for the weighted Bonferroni method [9], we estimated the conditional power for each SNP under all three genetic models. For each SNP, the result for the genetic model with highest power was selected and the lower powered results (without evaluating the FBAT statistic p-value) were discarded. This resulted in 500,000 SNPs/power estimates across the three genetic models, that were ranked overall by power and evaluated for association using weighted Bonferroni significance levels. The weighted Bonferroni significance levels were computed in the same way as previously described. We then compared the power obtained from the weighted method to standard Bonferroni correction, which computed the FBAT statistic under all three genetic models at each SNP, thus requiring a correction for 1.5 million comparisons (500,000 markers * 3 genetic models) and an FBAT p-value <3.3×10^−8^ for significance (i.e., 0.05/1,500,000). For simplicity, we ran these simulations for 2000 trios.

The results of the second set of simulations are displayed in Table 2. The data are presented in an identical format to the simulations under the additive model (including partitioning parameters of K = 7 and r = 2), except that column 1 reflects the “true” underlying genetic model rather than the number of trios.

**Table 2:** Power for 2000 trios and 500K markers, using mating type ratio equation R4, under an “unknown” genetic model.

True Gen.	Odds	MAF	Independence scenario		LD scenario (DSL only)		LD scenario (DSL+)	
Model	Ratio		Weighted	Standard	Weighted	Standard	Weighted	Standard
**Add.**								
	1.25	0.1	0.033	0.001	0.019	0.000	0.074	0.005
		0.2	0.140	0.008	0.085	0.002	0.265	0.055
		0.3	0.175	0.022	0.109	0.007	0.320	0.122
		0.4	0.137	0.029	0.083	0.007	0.305	0.174
	1.375	0.1	0.140	0.026	0.098	0.008	0.256	0.092
		0.2	0.414	0.171	0.316	0.085	0.623	0.430
		0.3	0.537	0.332	0.373	0.166	0.777	0.644
		0.4	0.532	0.404	0.376	0.241	0.793	0.711
	1.5	0.1	0.354	0.183	0.281	0.107	0.546	0.385
		0.2	0.790	0.646	0.669	0.466	0.928	0.876
		0.3	0.910	0.844	0.802	0.694	0.984	0.967
		0.4	0.916	0.878	0.817	0.742	0.985	0.973
**Dom.**								
	1.5	0.1	0.207	0.099	0.135	0.053	0.360	0.230
		0.2	0.376	0.257	0.271	0.154	0.597	0.490
		0.3	0.306	0.218	0.200	0.129	0.522	0.443
		0.4	0.145	0.104	0.072	0.046	0.263	0.204
	1.75	0.1	0.760	0.690	0.642	0.548	0.896	0.856
		0.2	0.937	0.910	0.862	0.808	0.988	0.979
		0.3	0.906	0.868	0.821	0.758	0.967	0.951
		0.4	0.693	0.624	0.577	0.503	0.830	0.784
	2	0.1	0.989	0.984	0.970	0.959	1.000	0.999
		0.2	1.000	0.999	0.999	0.999	1.000	1.000
		0.3	0.997	0.995	0.993	0.992	0.999	0.998
		0.4	0.965	0.950	0.935	0.911	0.987	0.982
**Rec.**								
	2	0.1	0.002	0.000	0.002	0.000	0.002	0.000
		0.2	0.011	0.005	0.008	0.003	0.019	0.007
		0.3	0.217	0.165	0.147	0.104	0.335	0.267
		0.4	0.767	0.723	0.657	0.598	0.887	0.867
	2.25	0.1	0.003	0.000	0.002	0.000	0.006	0.000
		0.2	0.039	0.014	0.029	0.010	0.057	0.029
		0.3	0.562	0.463	0.450	0.373	0.704	0.620
		0.4	0.971	0.959	0.949	0.927	0.991	0.985
	2.5	0.1	0.005	0.000	0.004	0.000	0.007	0.000
		0.2	0.103	0.053	0.068	0.036	0.155	0.087
		0.3	0.850	0.784	0.783	0.709	0.926	0.884
		0.4	0.997	0.995	0.995	0.991	1.000	1.000

Estimated power levels to detect the DSL using 2000 trios, assuming a 10% disease prevalence. The significance level is set to 5%. For the weighted Bonferroni method (Weighted), the partitioning parameters are *K* = 7 and *r* = 2. “Under True Gen. Model”, Add. refers to the scenario where the true (but “unknown”) model is additive (as the results are analyzed using all three genetic models). Similar scenarios are provided for the dominant (Dom.) and recessive (Rec.) genetic models. MAF denotes minor allele frequency. The power reflects the proportion of times the p-value of the DSL (Independence scenario and LD scenario (DSL only)) or a SNP in LD with the DSL (LD scenario (DSL+)) met the weighted Bonferroni (Weighted) or standard Bonferroni corrected (Standard) significance level. The standard Bonferroni correction adjusts for 1.5 M comparisons (500 K markers ^*^ 3 genetic models).

For the additive model, in comparison to the simulations where the genetic model is known, the power estimates tend be slightly lower. In the independence scenario, for an odds ratio of 1.5 and MAF of 0.2, when the genetic model is known, the weighted Bonferroni method has 91% power versus 85% for the standard, whereas, when the genetic model is unknown, the power estimates are 80% and 57%, respectively. However, our new method seems much more robust to analysis under multiple models in comparison to the standard correction. For an effect size of 1.5, the power loss in the unknown model ranges from 7 to 15%, depending on MAF, while power loss under the standard method ranges from 15 to 63%. Similar observations are made for the power comparisons between the weighted and standard methods for the LD scenarios. The overall power is reduced relative to the situation where the generating genetic model is known, but the difference in power between the weighted and standard methods is more striking. In comparing the independence scenario to the LD scenarios, the patterns observed when the genetic model is known hold here as well: when LD is present and the DSL or SNPs in LD with the DSL are considered, the power is highest, followed by the independence scenario. The lowest overall power is noted when LD is present and only the DSL is examined for significant association. In summary, while the overall power drops, the benefits of our methodology versus the standard are more pronounced when the genetic model is unknown and multiple analyses are conducted.

In comparing our method with weighted Bonferroni significance levels to the standard under dominant and recessive models, our procedure consistently demonstrates greater power, regardless of the degree of LD, effect size, or MAF. However, under a recessive model, a MAF of 0.3 or greater is required to achieve adequate power for the range of effect sizes that we examined (OR = 2–2.5).

Overall, our new methodology has the greatest impact for the low to moderately powered markers. For SNPs with standard Bonferroni power estimates ranging between 40% and 70%, the new method generally boosts power by an absolute difference of 10–15%, potentially providing marginally powered SNPs with a better chance of detection.

### Summary

Our simulation studies illustrate that the application of the proposed testing strategy is not limited by the number of trios analyzed, the degree of correlation among SNPs, the genetic model, or the size of the genetic effect. When standard approaches fail to provide sufficient power, the proposed testing strategy maintains acceptable power levels for small to moderate effect sizes (n = 2000) for the additive generating models, and moderate effect sizes under the dominant or recessive models or designs with fewer trios (n = 500–1000). As a general rule of thumb, our simulation experiments suggest that the testing strategy achieves optimal power levels for partitioning parameters of K = 7 and r = 2 for 500,000 markers, though power estimates were similar for K = 5–10 and r = 2–3. A comparison of the achieved power levels for differing number of trios and various genetic models illustrates that the impact of the multiple testing problem on a genome-wide association study can be minimized by the use of the proposed testing strategy.

### Data Analysis: A Genome-Wide Screen of Children Asthmatics

Asthma is a complex respiratory disorder, likely due to both genetic and environmental influences that affect the developing respiratory system. Asthma has been shown to have substantial heritability [27],[28],[29] and a comprehensive review of the literature in 2003 reported more than 200 studies with an association between asthma and its related phenotypes [30].

Thus, we applied our methodology to a family-based genome-wide association study of asthma. The families were originally recruited through the Childhood Asthma Management Program (CAMP) [31] Genetics Ancillary Study. All of the families were ascertained through asthmatic probands between 5 and 12 years old with mild to moderate asthma. All of the probands are affected, making it impossible to apply methodologies that require phenotype variation.

SNP genotyping was performed using Illumina HumanHap 550v3 arrays. Of 547,645 SNPs, 2.5% were removed during data cleaning due to genotype completion rates <95%, parent-offspring Mendelian errors, or because the assay sequence could not be aligned to one genomic locus, which resulted in 534,290 autosomal markers for analysis. Genotyping was conducted on 1215 subjects in 422 families. After removing 43 subjects with inadequate data, 1172 subjects comprising 403 families were analyzed. We applied the new power rank-weighting methodology, under an additive genetic model, to all 534,290 SNP, using equation R4 (Equation 6) to estimate genetic effect sizes, which had consistently had the highest power in the simulation studies. The power rankings were used to individually weight the family-based association test (also assuming an additive model) for each marker, using the method of Ionita-Laza et al. [9]. Table 3 displays the results for the CAMP data analysis. Based on the results of the simulation studies, the partitioning parameters of K = 7 and r = 2 were used.

**Table 3:** CAMP results: SNPs meeting genome-wide significance at α = 0.05.

Marker	MAF	H-W Equil.	Num. Info. Families	FBAT p-value	Power Rank	Required Significance Level
rs10863712	0.471	0.813	275	0.0032	1	0.005
rs1294497	0.490	0.882	276	0.0047	2	0.005

Results of the CAMP analysis with 402 families, 534,290 SNPs, assuming an additive mode of inheritance. Num. Info. Families indicates the number of families that were informative (i.e., at least one parent was heterozygous) for the marker of interest, and MAF denotes minor allele frequency. Markers with fewer than 20 families were removed from the analysis, as the asymptotic properties required for the test statistic may not hold. The power ranks are obtained from the conditional power of the test, calculated using our new technique with mating type ratio equation R4. The required significance level is obtained using the Ionita-Laza method [9] with *K* = 7, *r* = 2, and α = 0.05.

From the analysis, two SNPs were identified as genome-wide significant with a global alpha level of 0.05. These SNP were also the top two by power. Thus, the Top K Method by Van Steen et al. [8], with a modest choice of ‘Top’ markers selected for analysis, would have also detected these SNPs. However, the weighted Bonferroni method by Ionita-Laza et al. [9] allows for the evaluation of all SNP. Most strikingly, neither of these SNPs would have been detected after standard Bonferroni [7] or FDR-type [32] correction. These significant markers reside on chromosomes 1 (rs10863712) and 14 (rs1294497). In both markers, the minor allele is over-transmitted to the affected proband. These markers are currently under further study. These results provide proof of concept for our new method in that the top-ranked markers by power also showed evidence of association, strongly suggesting consistency of association in the independent population level and family level components of family-based data.

## Discussion

With the current genotyping capabilities, genome-wide association studies have become a reality. In order to utilize the wealth of SNP data obtained in such studies to identify genes for complex diseases, new statistical approaches are needed that can handle the multiple comparisons problem on an increasingly large scale. For population-based studies, multi-stage designs have been suggested. In each stage of the design, the “most promising” SNPs (top 1–10% of all genotyped SNPs) are pushed forward to the next level in which they are genotyped in another sample. Overall significance is established by combining the evidence from all stages into a single analysis. While this is a cost-effective approach, it is not as powerful as genotyping all subjects [33].

Testing strategies that use the same data set for genomic screening (i.e., rank-weighting) and testing [8],[9],[10],[11] establish genome-wide significance within one data set. They usually identify only a handful of SNPs (typically fewer than 20) which are then genotyped in other studies in order to generalize the significant findings [34],[35]. In contrast to multi-stage designs, genotyping the identified SNPs in other samples does not serve the purpose of establishing genome-wide significance. The effects of study heterogeneity are thereby limited. However, thus far, such testing strategies have only been available for the small subset of family-based studies in which the primary phenotype is quantitative, but not for the most popular family design, the classical trio design. The lack of phenotypic variation has prevented the genetic effect size estimation by the conditional mean model in the rank-weighting step.

In this manuscript, we have developed an approach that makes such testing strategies available for the commonly used TDT design. Our simulation studies show that our method outperforms standard methodology substantially. The effect size estimators that we suggest allow for the assessment of the genotype relative risk at a population level in ascertained family samples. In contrast to association tests for affected-only designs in population-based studies [12],[13],[14], here it is possible to estimate the genetic effect size independent of the unknown allele frequency. While we have discussed only the construction of such effect size estimators for the trio design, the concept of identifying probability ratios of mating types that depend on the genetic effect size, but not on the unknown allele frequency, is generally applicable to all family-based designs.

### URL

The testing strategy as well as the corresponding power and sample size calculations has been fully implemented in the software package *PBAT*, which is freely available at http://www.biostat.harvard.edu/˜clange/default.htm [36],[37].
